# The Impact of* Plasmodium* Infection on Placental Histomorphology: A Stereological Preliminary Study

**DOI:** 10.1155/2019/2094560

**Published:** 2019-03-03

**Authors:** John Ahenkorah, Patience B. Tetteh-Quarcoo, Mercy A. Nuamah, Bethel Kwansa–Bentum, Hanson G. Nuamah, Bismarck Hottor, Emmanuel Korankye, Magdalene Torto, Michael Ntumy, Fredrick K. Addai

**Affiliations:** ^1^Department of Anatomy, School of Biomedical and Allied Health Sciences, College of Health Sciences, University of Ghana, Ghana; ^2^Department of Medical Microbiology, School of Biomedical and Allied Health Sciences, College of Health Sciences, University of Ghana, Ghana; ^3^Department of Obstetrics and Gynaecology, School of Medicine and Dentistry, College of Health Sciences, University of Ghana, Ghana; ^4^Department of Animal Biology and Conservation Science, University of Ghana, Legon, Ghana

## Abstract

**Background:**

Malaria during pregnancy may threaten the mother's health and cause serious structural damage to the internal architecture of the placenta, which subsequently affects the pregnancy outcome. A better understanding of the impact of malaria parasites on the placenta morphology is crucial for better management of pregnant women and their babies.

**Aim:**

To assess by stereology the histomorphology of selected placental structures in placenta malaria compared with normal placentae at term.

**Method:**

A total of 10 placentae comprising 5 controls and 5 cases were selected from 50 placentae that were collected at term (38 weeks ± 2 weeks) from the maternal delivery suit of Korle-Bu Teaching Hospital in Accra, Ghana. Blood from the placentae was collected for both rapid diagnostic test and microscopic examinations. Samples collected were examined for* Plasmodium* parasites, after which they were classified as study group (*Plasmodium *positive) or control (*Plasmodium *negative). Stereological quantification using systematic uniform random sampling technique with test point and intersection counting of photomicrographs were employed to estimate the mean volume densities of syncytial knots, syncytial necrosis, foetal capillaries, and intervillous spaces of the placentae on a total of 1,600 photomicrographs.

**Results:**

Out of the fifty placental samples from the maternal side tested for* Plasmodium, *six representing 12% were found to be infected with the parasite by both rapid diagnostic test and microscopy. On stereological assessment, the mean volume density of syncytial knots was significantly higher in the placental malaria group compared with the control placentae at term (P = 0.0080), but foetal capillaries (P = 0.7813), intervillous spaces (P = 0.8078), and syncytial necrosis (P = 0.8249) were not significantly different.

**Conclusion:**

This preliminary result indicates that placental malaria may cause significant increase in the syncytial knots but not foetal capillaries, intervillous spaces, or syncytial necrosis. This finding signifies early maturation of the placenta and may be crucial in understanding perinatal outcomes.

## 1. Introduction

Malaria remains a complex and overwhelming health problem known to contribute significantly to maternal and infant mortality. Pregnant women, in particular, are more susceptible to malaria than the general population [1]. Studies attribute the increased susceptibility to the lack of immunity to pregnancy-specific isolates that sequester in the placenta [2, 3]. Although the mechanism for placental parasitization has not been fully understood, certain parasite strains have been on the increase in pregnant women, leading to the suggestion that these strains have the ability to adhere to the chondroitin sulfate A (CS-A) receptors on the syncytiotrophoblast; thus they are able to selectively infect the placenta [4, 5].

Placenta malaria, the accumulation of* Plasmodium*-infected erythrocytes in the intervillous space of the placenta, is one major feature of malaria during pregnancy. Maternal peripheral parasitemia is an indication of malaria during pregnancy. However, it has been observed that while peripheral parasitemia may sometimes remain below microscopic detectable levels, these parasites are detectable in the placenta [5, 6]. The accumulation of the malaria parasite in the intervillous spaces can alter the sensitive morphology of the placenta [7, 8].

Placental malaria has been associated with different adverse pregnancy outcomes. Some studies reported an association between malaria and the risk of stillbirth [9, 10]. Other studies have associated placental malaria with reduced birthweight [11, 12]. Another study demonstrated how placental malaria can cause foetal growth restrictions. [13]. Although many studies have reported different prenatal complications associated with placental malaria, there are less reports concerning the mechanism and factors that lead to the adverse outcome.

Stereology is the first choice for obtaining three-dimensional data from two-dimensional profiles [14]. It offers a toolkit of transparent sampling rules and simple estimation tool which allows 3D quantities (total and average volumes, surfaces, lengths, numbers, etc.) to be calculated from 2D images appearing on slice planes [14]. Modern stereology and design-based methods permit the quantitative description of morphology [14]. When applied to placentae in normal and abnormal pregnancies, it has proven to be of great value for challenging earlier misconceptions about interpretation of the growth, morphogenesis, adaptation, and functioning at the whole-organ level [15].

Despite the public health importance of placental malaria, its impact on the placenta histomorphology has not been fully understood. A better understanding is crucial in developing an effective intervention for the control of placental pathology associated with placental malaria. The objective of this study was to assess by stereology the histomorphology of selected placental structures in placental malaria compared with normal placentae at term.

## 2. Materials and Methods

This was a case control study, involving a purposive sampling of the postpartum placenta of 50 patients. Between 2 and 3 ml of blood from the maternal as well as the foetus side of the placenta was collected and kept in K_2_EDTA tubes (BD Vacutainer®, Becton, Dickinson & Company (BD), UK) for both* Plasmodium* rapid diagnostic test (RDT) and Giemsa stained smear microscopic examinations, after which it was classified as cases (study group) or controls. The study group was made up of placental samples that were found to be infected with the* Plasmodium*, while the control group was comprised of* Plasmodium *negative placenta samples.

### 2.1. Giemsa Stained Thick and Thin Film Preparation

A grease free glass slide with frosted end (Sigma-Aldrich Corp®, St Louis, USA) was used for the thick and thin films. For thin film, 2 *μ*l of a thoroughly mixed whole blood sample (some of which has earlier been used for the RDT) was pipetted and put in the middle of the slide. Using a fine edge glass slide spreader, the blood was spread rapidly at an approximate angle of 45° to get a thin monolayer [16].

For thick film, 5 *μ*l of the blood was pipetted and placed closed to the frosted end of the same slide having the thin film. The 5 *μ*l blood drop was spread circularly to obtain a diameter of between 1 and 2 cm smear. The films were air-dried for three hours at room temperature, after which the thin film was fixed in absolute methanol. Both the thick and thin films were flooded with 3% Giemsa for 30 minutes, followed by washing under running tap water and air-drying. The stained smears were then examined under a light microscope (Olympus Optics Ltd.®, UK) with oil immersion objective lens with the* Plasmodium* positive or negative smears leading to classification as case (study group) or control, respectively.

### 2.2. Rapid Diagnostic Test (RDT)

An immunoblot test kit (Immunetics Inc.) was used for the rapid diagnostic test (RDT). Following the manufacturer's instruction, a drop of the blood collected from the placenta was placed on the sample column of the test kit, after which four drops of the lysing buffer were added, with the results interpreted as RDT positive or RDT negative.

### 2.3. Tissue Slicing and Processing

A total of ten placentae comprising 5 controls and 5 study group were selected for further examination. After weighing the placenta and recording the volume by liquid displacement, each placenta was then cut into four equal quadrants and by choosing a random starting point, a tissue was sampled full-depth (from chorionic plate to basal plate) from each quadrant concentrically from the periphery to the centre.

Placenta samples of about 1cm x 1 cm x 1cm cubes were fixed in 10% buffered formalin, pH 7.29, after which the tissues were dehydrated through graded series of ethanol (from 70% through to 95%). The placenta tissues samples were further dehydrated with steps of absolute alcohol, after which they were cleared in two steps of xylene. Tissues samples were infiltrated with molten wax and embedded to form blocks for microtome sectioning. Each placental tissue sample from each quadrant was embedded in one wax block.

### 2.4. Sectioning of Placenta Tissue Samples

**Figure 1:**
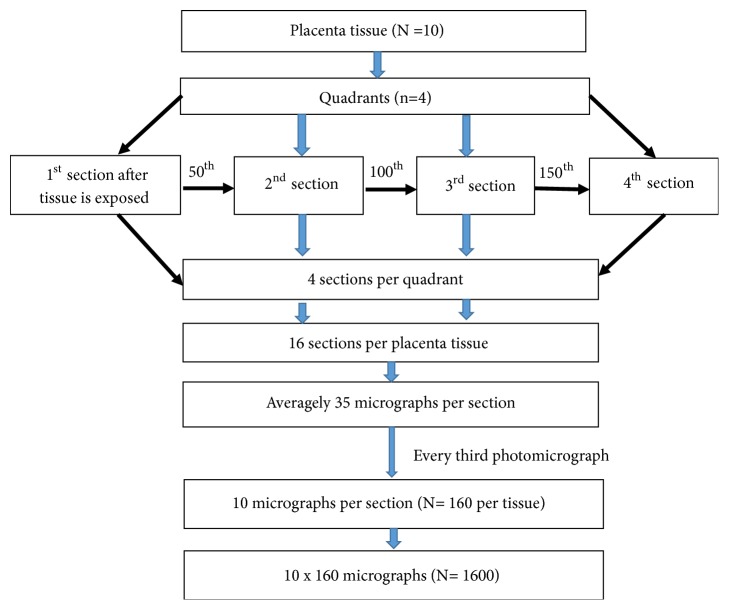
Summary of stereological systematic random sampling.

The wax-blocked placental tissues were initially trimmed at a thickness of 10 micrometres (*μ*m) to expose the whole profile of the tissue using Leica microtome (Leica RM 2125, Germany). The tissues were then sectioned at a thickness of 5 *μ*m and four sections were selected systematically and stained with Haematoxylin and Eosin (H&E). The 1^st^, the 50^th^, the 100^th^, and 150^th^ tissue sections were selected from each block (Figure 1). A total of 16 sections were collected for one placenta tissue. The sections of the placental tissues were picked onto the glass slide (76 mm x 26 mm×1mm). A total of 160 placental sections were pooled from the 10 placenta tissues for histomorphometrical studies.

### 2.5. Stereological Studies

#### 2.5.1. Sampling of Photomicrographs of Placenta Sections

The section was brought to focus with x40 objective lens under optical light microscope (Leica Galen III, catalogue no. 317506, serial no. ZG6JA4). The microscope field was directed to one end of the slide (from one corner of the basal plate). An eyepiece lens of the microscope was then replaced with a digital microscope eyepiece (Lenovo Q350 USB PC Camera), which was connected to a desktop computer (HP Compaq dx2300 Microtower). The stage was moved two microscope stage units in the* x*-axis and two microscope stage units in the* y*-axis and then photomicrograph taken. The stage was then moved in the opposite direction in the* x*-axis but in the same direction on the* y*-axis for the next micrograph to be taken. These movements were repeated till the whole area of the placenta section was covered. The images of all placenta micrograph profiles which were encountered during these movements were captured using x40 objective and the digital camera. The average number of micrographs taken was 35 per placental slide. All the micrographs obtained using the 16 sections per placenta tissue were pooled together and ten photomicrographs were selected using systematic random sampling, where every third photomicrograph was selected from the first random selected photomicrograph for stereological studies. A total of 160 sample micrographs were selected systematically per placenta tissue (Figure 1).

**Figure 2:**
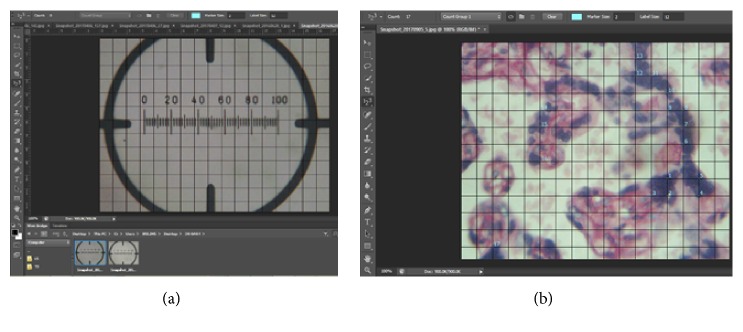
(a) Grid being calibrated using Adobe Photoshop. (b) Point counting of syncytial knot using Adobe Photoshop.

In total, 1,600 photomicrographs were systematically selected for point counting in the stereological grid (Figure 2). In addition, snapshots of microscope stage graticule were taken at the same magnification. The micrograph of the graticule was used for calibrating the grid which was used for the stereological study (Figure 2(a)). The H&E sections were used for counting syncytial knot, syncytial necrosis, intervillous space, and foetal capillaries.

#### 2.5.2. Stereological Study of Placental Photomicrographs

The volume densities of the syncytial knot, foetal capillaries, villi syncytial necrosis, and intervillous space were determined using point counting with Cavalieri principle [17]. A stereological grid consisting of uniformly spaced points, 1cm × 1cm, was superimposed over each micrograph of placenta section using Adobe Photoshop CS6 Extended (trial version 13.0.1) software. The number of tests at the intersections of the grid points was counted as shown in Figure 2(b). Volume densities of syncytial knot, foetal capillaries, villi syncytial necrosis, and intervillous space were calculated using the following equation:

(1) $$\begin{array}{c} Vv=\frac{ƩP\times \left(a/p\right)\times t}{M^{2}} \end{array}$$

where* Vv* indicates volume density, *Ʃ*P is the sum of all test points encountered, (*a/p*) is the area per point of the stereological grid,* t* is the thickness of the section, and* M* is the linear magnification [18].

## 3. Results

Out of the total of 50 placenta samples that were tested for* Plasmodium* parasites, six (12%) were found to be infected with* Plasmodium* parasite by both RDT and microscopy, while the rest (44, 88.3%) were negative for* Plasmodium *by both diagnostic methods. Therefore, the 12% (*Plasmodium *positive placenta samples) became the study group, while the 82% (*Plasmodium *negative placenta samples) became the control group.

**Figure 3:**
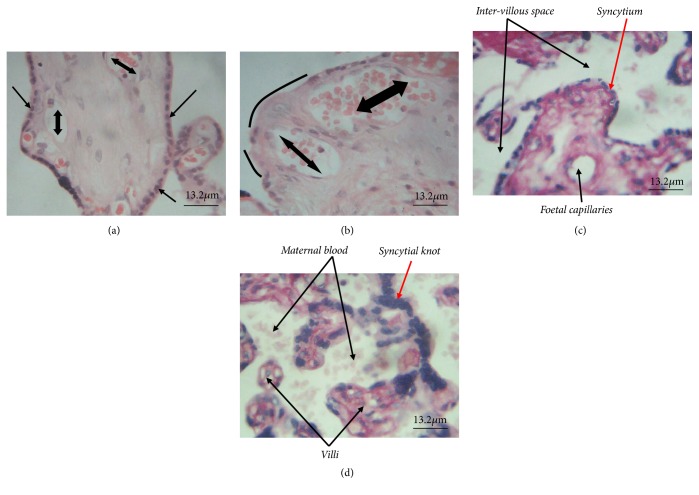
Histomorphological assessment of placental tissues (H&E stained). (a) Placental tissue of control sample with normal syncytium indicated with* single head arrow*. (b) Placental tissue of case sample with syncytial necrotic areas indicated with* curvy lines*.* Double head arrows* in (a) and (b) indicate foetal capillaries. (c) and (d) show internal architecture of the placenta tissues in control and study groups, respectively; notable among them is the normal syncytium in (c) compared to the significant number of syncytial knots seen in (d), indicated by red arrows.

With regard to histomorphological assessment, syncytial necrosis, where the syncytium is shaved off (as shown with curved lines in Figure 3(b)) compared to normal syncytium (shown with single head arrow in Figure 3(a)), was among the structural damage to the internal architecture of the placenta observed in the photomicrograph of the placenta tissue. Also, it was observed that clumps of syncytiotrophoblast nuclei forming the syncytial knots were significantly high in number among plasmodium-infected placentae (Figure 3(d)) compared to the control group (Figure 3(c)).

**Figure 4:**
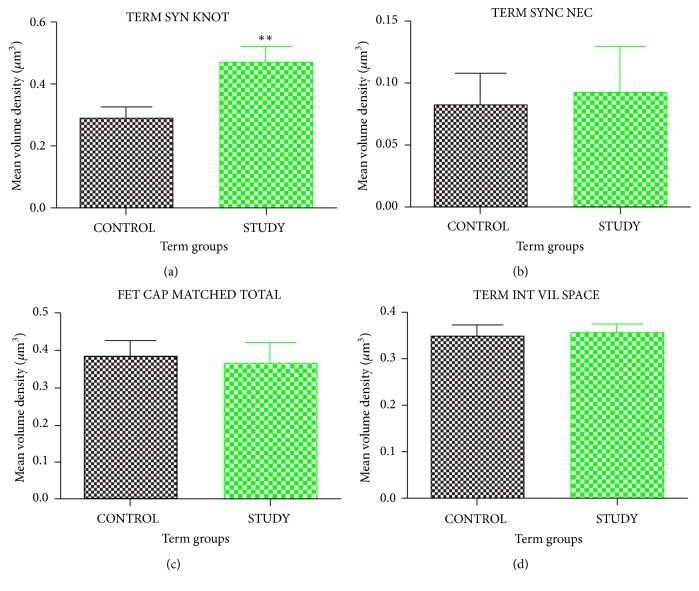
Unpaired t-test analysis of placental parameters at term, comparing healthy (control) and* Plasmodium*-infected placenta (study group). (a) Volume density of syncytial knot. (b) Volume density of syncytial necrosis. (c) Volume density of foetal capillaries. (d) Volume density of intervillous space. SYN KNOT represents syncytial knot, SYNC NEC represents syncytial necrosis, FET CAP represents foetal capillaries, and INT VIL SPACE represents intervillous space. Values are expressed as mean ± SEM. P value represents significance level for unpaired* t*-test for time course assessment for term group comparison with *∗*=P<0.05, *∗∗*=P<0.01, and *∗∗∗*=P<0.001.

The unpaired* t*-test analyses of the placenta at term reveal no significant difference for volume density of intervillous space, foetal capillaries, and syncytial necrosis, with respective recorded P values of 0.8078, 0.7813, and 0.8249, between malaria-infected placenta and the control group. Nevertheless, the mean volume density of syncytial knots was significantly increased in the term study population when compared to the term control group (Figure 4). P value of 0.0080 was recorded with regard to volume density of syncytial knots with mean increase of 0.178 ± 0.02 for the term study group (Figure 4).

## 4. Discussion

The prevalence (12%) observed in this study was lower than a previous study conducted in Ghana [19], which reported 35.9% malaria parasites in the placentae of pregnant women. A different study conducted in Ghana reported similar prevalence (36.3%) among the pregnant women [20]. These differences may be due to variations in community-acquired immunity, sociodemographic characteristics of the study population, use and resistance to malaria chemoprophylaxis, and the diagnostic tools employed in the detection of the parasite [21–23]. Also, the difference in time for these earlier studies (conducted in 2009) in Ghana [19, 20] and the current one (conducted in 2018), spanning almost ten years, could have accounted for the decline in placental malaria prevalence. This is because within this time there have been differences in the implementation of malaria treatment, prevention, and control strategies, such as intermittent preventive treatment (IPTp-SP) and the mass distribution of insecticide-treated bed nets in Ghana, with a lot of attention on pregnant women. This reason is supported by the observation made by Hommerich* et al*. [24] who reported on a decline of placental malaria in southern Ghana (where the current study was carried out) between 2000 and 2006 after the implementation of the IPTp-SP in pregnancy. Similar report has been made by Bouyou-Akotet* et al*. [25] in Gabon and Kayentao* et al*. [26] in Mali.

However, similar low placenta malaria prevalence of 12% has been recorded in a study conducted in Tanzania, which involved 984 placental samples [27]. Other studies have also reported comparable prevalence of 11% in Agogo community, Ashanti Region, Ghana, and 8% in Rufiji district, Pwani Region, Tanzania [24, 28].

Nevertheless, the 12% prevalence of placental malaria suggests that pregnant women are still in the clutches of malaria, despite present control measures like insecticide-treated nets (ITN) and intermittent preventive treatment in pregnancy (IPTp), among others [24, 29].

The findings of this study were consistent with other studies that also reported increased syncytial knots in the* P. falciparum*-infected placentae [7, 30]. The mechanism accounting for the increase in the volume density of syncytial knot is not clear. Perhaps the increase of syncytial knots counted in this preliminary study could be due to a series of events such as blocking of the spiral arterioles by infected erythrocytes, leading to oxidative stress, thereby leading to apoptotic events at the syncytium. However, it has been reported in another study that significant increase in syncytial knot formation in placental villi indicates the disturbance in the hormonal factors, which may probably lead to altered blood flow [31]. A review article suggested that the pathology that develops in the placenta and the adaptations the placenta undergoes to mitigate this pathology may influence the later health of the mother and the baby [32].

The strength of our preliminary study is the use of stereology to count placental internal architecture (syncytial knots, syncytial necrosis, foetal capillaries, and intervillous spaces) in 1, 600 photomicrographs of placentae affected by malaria compared with controls.

A limitation, however, is the inability to tell how long the parasite has been in the placenta. It may be interesting to quantify by stereology these internal structures of the placenta in early exposure to the parasite and that of late infection in future research. Another limitation worth mentioning is the low number of placentae samples used in this preliminary study, since this could possibly account for the percentage of* plasmodium*-infected placentae being lower than what other studies have reported, as well as the nonsignificant differences observed in some internal architecture of the* plasmodium*-infected placentae compared to the uninfected ones.

## 5. Conclusion

*Plasmodium* infections during pregnancy have adverse effect on the internal architecture of the placenta that may also explain perinatal outcomes. Our preliminary results add to the knowledge that placental malaria may cause significant increase in the syncytial knots, which signifies early maturation of the placenta.

## Data Availability

The hard copies and electronic data used to support the findings of this study are available from the corresponding author upon request: Dr. Patience B. Tetteh-Quarcoo's e-mail: patborket2002@yahoo.com or pbtetteh-quarcoo@ug.edu.gh.
